# Recuperating Biopharmaceutical Aspects of Amphotericin B and Paromomycin Using a Chitosan Functionalized Nanocarrier via Oral Route for Enhanced Anti-leishmanial Activity

**DOI:** 10.3389/fcimb.2020.570573

**Published:** 2020-10-15

**Authors:** Shabi Parvez, Ganesh Yadagiri, Archana Karole, Om Prakash Singh, Anurag Verma, Shyam Sundar, Shyam Lal Mudavath

**Affiliations:** ^1^Infectious Disease Biology Laboratory, Institute of Nano Science & Technology, Mohali, India; ^2^Department of Biochemistry, Institute of Science, Banaras Hindu University, Varanasi, India; ^3^School of Pharmaceutical Sciences, Institute of Foreign Trade and Management (IFTM) University, Moradabad, India; ^4^Teerthanker Mahaveer College of Pharmacy, Teerthanker Mahaveer University (TMU), Moradabad, India; ^5^Infectious Disease Research Laboratory, Department of Medicine, Institute of Medical Sciences, Banaras Hindu University, Varanasi, India

**Keywords:** amphotericin B, oral delivery, paromomycin, visceral leishmaniasis, solid lipid nanoparticles

## Abstract

The design and development of new pharmaceutical formulations for the existing anti-leishmanial is a new strategic alternate to improve efficacy and safety rather than new drug discovery. Herein hybrid solid lipid nanoparticles (SLN) have been engineered to direct the oral delivery of two anti-leishmanial drugs amphotericin B (AmB) and paromomycin (PM). The combinatorial nanocarriers consist of conventional SLN, antileishmanial drugs (AmB and PM) which have been functionalized with chitosan (Cs) grafted onto the external surface. The Cs-SLN have the mean particle size of 373.9 ± 1.41 nm, polydispersity index (PDI) of 0.342 ± 0.02 and the entrapment efficiency for AmB and PM was found to be 95.20 ± 3.19% and 89.45 ± 6.86 %, respectively. Characterization of SLN was performed by scanning electron microscopy and transmission electron microscopy. Complete internalization of the formulation was observed in Caco-2 cells. Cs-SLN has shown a controlled and slow drug release profile over a period of 72 h and was stable at gastrointestinal fluids, confirmed by simulated gastro-intestinal fluids study. Cs coating enhanced the mucoadhesive property of Cs-SLN. *The in-vitro* anti-leishmanial activity of Cs-SLN (1 μg/ml) has shown a maximum percentage of inhibition (92.35%) on intra-cellular amastigote growth of *L. donovani*.

## Introduction

Visceral leishmaniasis (VL), also known as kala-azar is a vector-borne infectious disease caused by an obligate intracellular protozoan parasite of *Leishmania* species and invades the reticuloendothelial system of spleen, liver, and bone marrow (Chappuis et al., 2007; Shrestha et al., 2018; Herrera et al., 2019; Martínez and Ruiz, 2019). VL is a serious public health issue in many tropical and subtropical regions of the globe and affects poor and malnourished people in developing and underdeveloped countries of Brazil, East Africa, and South-East Asia. VL is the most severe form of leishmaniasis, characterized by irregular bouts of fever, anemia, weight loss, hepatosplenomegaly, and is fatal if untreated (Sundar and Singh, 2016; Herrera et al., 2019). According to World Health Organization (WHO), 50,000–90,000 new VL cases reported globally and more than 95% of new VL cases reported in 10 countries: Bangladesh, Brazil, China, Ethiopia, India, Kenya, Nepal, Somalia, South Sudan, and Sudan.^1^ In, Indian subcontinent, VL is highly endemic in poor rural communities of Bihar state, which contributes nearly 80% of global disease burden and threats about 200 million people (Hasker et al., 2012). Although, chemotherapy is the mainstay of therapy for VL treatment and control, the clinical application of existing anti-leishmanial agents is limited due to serious toxic adverse effects and long term intravenous drug treatment options (Yadagiri and Singh, 2018). The emergence of drug-resistant parasites, HIV-VL co-infections, non-availability of effective proper vector control measures, and vaccine (s) against VL further complicated the disease control and treatment (Singh et al., 2016a; Ponte-Sucre et al., 2017). Pentavalent antimonials (sodium stibogluconate or meglumine antimoniate), the mainstay of therapy for VL have shown resistance and relapse in VL-infected patients of North Bihar, India, where VL endemicity is high (Mostafavi et al., 2019). Intravenous administration of amphotericin B for VL causes serious adverse effects including hypokalemia, myocarditis, and nephrotoxicity, which necessitates long hospital stays for close monitoring of patients and ultimately increases the cost of therapy. Aminoglycoside antibiotic, paromomycin for VL treatment causes nephrotoxicity and ototoxicity (Yadagiri and Singh, 2018). Several new VL treatment strategies have emerged during the past 10–15 years, but each has its serious own limitations. Therefore, better anti-leishmanial drug discovery is an immediate need for saving the lives of VL-infected people in endemic zones without showing any toxic adverse effects (Singh et al., 2016a).

During the last decade, nanotechnology-based drug delivery systems have been extensively used to improve the performance of drugs in treating several diseases with improved efficacy and safety. Some of the nanotechnology-based formulations have been approved by the U.S. Food and Drug Administration (FDA) for clinical application (Patra et al., 2018). Encapsulation of AmB in novel nanocarrier systems can target specific drug delivery in reticuloendothelial system of liver and spleen, where *Leishmania* parasites reside and replicate without showing nephrotoxicity (Gupta et al., 2012).

The need of the hour is to develop a delivery system, which annihilates the domination and inadequacy of currently available marketed formulations as well as deliver drug to macrophage-specific organs (spleen and liver). Combinational therapy is an attractive option to overcome the problems associated with the aforementioned drugs. Combinational therapy has an extensive array of advantages like shortening of the duration and lowering the cost of treatment, lowering the development of resistance. PM and antimonial used in short regimens were found more effective treatment as compared to conventional drugs (Monge-Maillo and López-Vélez, 2013; Singh et al., 2016b). Kun Shi et al. (2019) reported combinational therapy of gemcitabine and cis-platinum via thermo-sensitive copolymer micelles to target pancreatic cancer (Shi et al., 2019). Trinconi et al. (2014) reported the effective role of combination therapy of tamoxifen and AmB for cutaneous leishmaniasis gave good clinical as well as parasitological response (Trinconi et al., 2014). Hence, combinational therapy has ascertained to be more effective as compared to single-drug therapies.

The oral route is the most convenient and safest route along with the highest patient compliance, low cost, and lesser complication in comparison to other routes of administrations. Poor solubility and poor permeability of drugs are needed to be considered for the enhancement of oral bioavailability. To overcome the obstacle allied with oral drug delivery systems, nanosized or nanoscale (NP) particles could be considered as an excellent alternative to conventional drug delivery systems and generally used to augment the oral bioavailability of drugs. Solid lipid nanoparticles (SLN) have attracted the attention of our research group to develop a formulation with enhanced oral bioavailability of antileishmanial drugs.

SLN is a colloidal carrier (Vivek et al., 2007) which is a classically sphere-shaped average particle size found in the range of 50–500 nm (Chavan et al., 2013). SLN are composed of solid lipid core matrix which can solubilize lipophilic drug. The lipid core is become stable by surfactant (Vivek et al., 2007). SLN holds various advantages like high drug payload, long term stability, controlled release profile. Due to the unique characteristics of SLN, it could be used for delivery of both hydrophilic (PM) and hydrophobic (AmB) drugs, depending on the type of lipid, surfactant, and method of preparation (Mendonça et al., 2020).

Chitosan (Cs) is a polycationic naturally occurring bio-degradable, non-toxic, non-allergenic bio-polysaccharide derived from chitin which is abundant in nature (Jain et al., 2018). Cs is studied as the most efficient material for impending use on account of its exemplary bio-degradability, bio-compatibility, non-toxicity, antimicrobial activity, and is cost-effective. Cs exhibits escalating mucoadhesive properties that expedite and enhance the transport capacity of drug-loaded nanoparticles, their absorption across the GI tract, and eventually improve the drug bio-availability when administered orally (Singh et al., 2016b; Min et al., 2018). Surface modification of nanoformulation could have been used for the customization of therapeutic efficiency as well as the biodistribution profile (Jain et al., 2015).

In this study, Cs-coated SLN containing AmB and PM were developed and characterized to be tested as oral carriers for AmB and PM. NPs were assessed considering their physicochemical properties, stability on simulated GI fluids, and cytotoxicity over J774A.1 cells, cellular uptake studies, mucoadhesive property, and anti-leishmanial activity.

## Materials and Methods

### Materials

Chitosan (low molecular weight), 4′, 6-diamidino-2-phenylindole dihydrochloride (DAPI), fluorescein isothiocyanate (FITC), 3-(4, 5-dimethylthiazol-2-yl)-2,5-diphenyltetrazolium bromide (MTT) were purchased from Sigma-Aldrich (USA). AmB, glycerol monostearate (GMS), polyvinyl alcohol (PVA), Tween 80, polyethylene glycol 400 (PEG 400), 4% paraformaldehyde and cellulose dialysis tube (12 kDa) were procured from HiMedia Laboratories (India). RPMI 1640, heat-inactivated fetal bovine serum (HI-FBS), penicillin-streptomycin, rhodamine-phalloidin, trypsin 0.25%, glass-bottom dishes and phosphate buffer saline (PBS; pH 7.4) were purchased from ThermoFisher Scientific (India). Sodium taurocholate, paromomycin (PM) were obtained from SRL (India).

### Parasite and Cell Line

*Leishmania donovani* (LEM 138) parasites were cultured in M199 medium supplemented with antibiotics (100 U/ml penicillin, 100 μg/ml streptomycin) and 10% heat-inactivated fetal bovine serum (HI-FBS), maintained at 26°C BOD incubator. J774A.1 macrophage cells were cultured in RPMI-1640 supplemented with penicillin (100 U/ml) and streptomycin (100 μg/ml) and HI-FBS (10%), maintained at 37°C and 5% CO_2_ incubator.

### Fabrication of Unmodified/Surface-Modified Drug-Loaded Solid Lipid Nanoparticle

AmB and PM loaded SLN (DSLN) were prepared by the emulsion solvent evaporation method (Liu et al., 2011). Briefly, GMS (150 mg), AmB (50 mg) were dispersed in 5 ml of ethanol and soy lecithin (40 mg), heated to 60°C to compose the organic phase. The aqueous phase was formulated simultaneously by the addition of PVA (0.5% w/v), PEG 400 (1.5% w/v), and PM (20 mg), in double-distilled water (20 ml) maintained at 60°C. The organic phase was added drop by drop into aqueous phase with constant stirring at 1000 rpm, leading to the formation of an emulsion. The emulsion was then released to another aqueous phase; 1% Tween 80 (w/v) and 1% of PEG 400 (w/v) and stirred continuously at 4°C allowing the solidification of SLN. The resulting suspension was centrifuged at 11,000 rpm for 30 min (Avanti JXN-30 Beckman Coulter US) to collect the pellet, which was washed thrice with Milli-Q water. Mannitol (2% w/v) was added as a cryoprotectant and the resulting dispersion was freeze-dried (FDUT-12003, Republic of Korea) to get the dried DSLN. Formulated DSLN coated with Cs (Cs-SLN) were prepared by incubating the freeze-dried DSLN with chitosan (0.1% w/v) solution for 2 h at room temperature (Channarong et al., 2011; Guo et al., 2016). Plain SLN (PSLN) was prepared by a similar method without the drugs. FITC labeled Cs-SLN were prepared by adding FITC in the organic phase in the preparation methodology as described above.

### Encapsulation Efficiency and Drug Loading

Encapsulation efficiency and drug loading was determined by using a previously reported indirect method. Fmoc-PM complex was synthesized by solution-phase process for the determination of PM, Fmoc-Cl (4 mM) was added to acetonitrile and subsequently add to PM (0.5 mM) in borate buffer (pH 8.1) and stirred in completely dark conditions for few minutes. Ethyl acetate was used for extraction and the complex obtained was further used for the development of formulations.

After the preparation of DSLN, the supernatant was collected by centrifugation at 11,000 rpm for 30 min and it was used to measure the amount of Fmoc-PM complex at 315 nm by using fluorescence spectroscopy (F-4600 spectrophotometer, Hitachi, Japan) (Kumar and Bose, 2016) and AmB was determined using UV-Visible spectrophotometer (Shimadzu UV-2600) at 405 nm.

The entrapment efficiency and drug loading was determined by the following formulae.

(1) $$\begin{array}{l} \text{Entrapment efficiency }\left(\text{\%EE}\right)={\text{W}}_{\text{t}}-{\text{W}}_{\text{s}}/{\text{W}}_{\text{t}}*100 \end{array}$$

(2) $$\begin{array}{l} \text{Drug loading }\left(\text{\%DL}\right)={\text{W}}_{\text{t}}-{\text{W}}_{\text{s}}/{\text{W}}_{\text{l}}+{\text{W}}_{\text{t}}-{\text{W}}_{\text{s}}*100 \end{array}$$

where, W_l_ is the amount of lipid, W_t_ is the total amount of drug used, W_s_ the drug remaining in the supernatant.

### Characterization

#### Particle Size and Zeta Potential

Particle size, polydispersity index (PDI), and zeta potential of freshly prepared Cs-SLN were determined by using photon correlation spectroscopy (Zetasizer Nano ZSP; Model-ZEN5600, Malvern Instruments Ltd. UK) at 25°C. Concisely, Cs-SLN were sufficiently diluted with Milli-Q water (1:100) and placed in disposable cuvettes for the measurement of particle size and in disposable folded capillary cells for determination of zeta potential.

#### Morphology

Morphology of Cs-SLN was observed using scanning electron microscopy (SEM) and transmission electron microscopy (TEM). The Cs-SLN dispersion was drop-casted and then sputter-coated with gold under vacuum (JEC- 300 FC) and analyzed for SEM. For the TEM (JEOL 2100) analysis, the dispersion was drop cast onto 300 mesh carbon coated-copper grid and the surplus amount was taken off with filter paper and negatively stained with 2% w/v phosphotungstic acid (PTA) for contrast enhancements. Further, the samples were kept overnight for vaccum drying. TEM images were captured at an accelerating voltage of 200 kV, using Gatan camera software.

### FTIR Spectroscopy

Fourier transform infrared spectroscopy (FTIR) was performed to ascertain any chemical interactions present between GMS, AmB, PM, Cs, and Cs-SLN using Bruker vertex 70v spectrophotometer and were scanned at 400–4,000 cm^−1^ for at the resolution of 4 cm^−1^ and 64 scans performed for all samples.

### Powder X-ray Diffractometer (PXRD)

X-ray diffraction (XRD) patterns of AmB, PM, Cs, and Cs-SLN were obtained by using Bruker, D8 advance X-ray diffractometer outfitted with a Ni-filtered Cu Kα-radiation, λ = 1.541 Å (voltage 40 kV; current 30 mA). The diffraction pattern was recorded over a 2θ range of 10–70° at a scanning rate of 5° per min.

### Cellular Uptake

Confocal microscopy was used to study the internalization of FITC labeled Cs-SLN in Caco-2 cells to evaluate the oral uptake efficiency of Cs-SLN. Briefly, Caco-2 cells were seeded at an initial density of 10,000 cells per well in glass-bottom dishes in Dulbecco's modified Eagle's medium (DMEM) with 20% fetal bovine serum (FBS), 100 μg/ml streptomycin and 100 IU/ml penicillin, and placed in an incubator at 37°C for 48 h allowing the cells to adhere. The cells were treated with FITC labeled Cs-SLN for 2 and 6 h. The cells were washed thrice with 1X PBS (pH 7.4) and fixed with 4% paraformaldehyde for 20 min. The cells were washed thrice with PBS (pH 7.4) and stained with DAPI (1 μg/ml) for 4–5 min. Rhodamine-phalloidin was used to stain the cell membrane for 30 min. The cells were washed and images were acquired using a confocal laser scanning microscope (CLSM, LSM 880 NLO, Carl Zeiss, Germany).

### *In vitro* Cytotoxicity Study

MTT assay was used to evaluate the cytotoxicity of SLN on J774A.1 macrophage cells. In brief, 5 × 10^4^ cells/well were seeded into 96 well plates and were kept at 37°C and 5% CO_2_ environment, to adhere to cells for 24 h. The treatment to the cells was given with varying concentrations (3.12 μg/ml, 6.25 μg/ml, 12.50 μg/ml, 25 μg/ml, 50 μg/ml) of PSLN, Cs-SLN, and free drugs (AmB and PM) as well as the untreated cells were used as control. At a predetermined time, MTT reagent (20 μl of 5 mg/ml stock solution) was added followed by incubation for 4 h, under 5% CO_2_ environment, and at 37°C. After the removal of the medium, DMSO (100 μl) was added to dissolve formazan crystals. Cell viability was evaluated by was measuring the optical density (OD) of solutions by using a microplate reader (Infinite 200 PRO microplate plate reader) at 570 nm as well as 630 nm used as a reference wavelength (Khatik et al., 2014). Cell viability was calculated by applying the formula:

(3) $$\begin{array}{l} \text{Cell viability}=\text{Ab}{\text{s}}_{\text{s}}/\text{Ab}{\text{s}}_{\text{c}}*100 \end{array}$$

where, Abs_s_ is the absorbance of samples, and Abs_c_ is the absorbance of control.

### *In vitro* Release Study

Dialysis membrane method was used to evaluate the *in vitro* drug release study. The dialysis membrane (12 kDa) was soaked in Milli-Q water overnight and formulations were placed in dialysis membrane closed at both the ends with the clamps, which was then immersed in 250 ml phosphate buffer saline (pH 7.4) along with 1% Tween 80 and agitated at 150 rpm, 37 ± 1°C for 72 h. Aliquots were taken out at regular time intervals and replaced with fresh buffer to maintain the sink conditions. Samples were analyzed using UV-VIS spectroscopy (Shimadzu UV-2600) at 413 nm and by using fluorescence spectroscopy (Infinite 200 PRO microplate plate reader) for detection of PM at 312 nm.

### *In vitro* Simulated Gastrointestinal Fluid Stability Study

To evaluate the *in vitro* stability while passing through the GIT tract, Cs-SLN were placed in hydroxypropyl methylcellulose (HPMC) capsule and kept in individual dissolution baskets containing 500 ml medium (simulated gastric fluid (SGF; pH 1.6) and simulated intestinal fluids (SIF; pH 6.5) maintained at 37 ± 0.5°C under continuous stirring at 50 rpm. The SGF was prepared by adding sodium chloride (34.2 mM), lecithin (20 μM), sodium taurocholate (80 μM), pepsin (0.1 mg/ml), conc. HCl (q.s. pH 1.2) in 500 ml deionised water and SIF was prepared by adding lecithin (0.75 mM), sodium taurocholate (3 mM), sodium chloride (6.18 gm), sodium di-hydrogen phosphate (3.43 gm), and sodium hydroxide (q.s. pH 6.5) in 500 ml deionised water. Samples were taken out regularly and quantified using UV-VIS spectroscopy (Shimadzu UV-2600) at 408 nm (Klein, 2010).

### Mucoadhesive Property

Freshly prepared Cs-SLN were evaluated using the main glycoprotein in mucus i.e., mucin at different concentrations (0.1, 0.25, and 0.5% w/v), in 0.02 M phosphate buffer pH 6.8. Cs-SLN dispersion was incubated with mucin solutions for 30 min in a shaker at 70 rpm, 37°C. Particle size, PDI, Zeta potential were recorded before and after the incubation period (Pauluk et al., 2019).

### *In vitro* Anti-leishmanial Activity of Cs-SLN Against *L. donovani* Amastigotes

*In vitro* anti-leishmanial activity of Cs-SLN, AmBisome, and AmB were tested against intracellular amastigotes of *Leishmania* parasite. Briefly, macrophage cells (J774A.1) (2.5 × 10^5^ cells/ml) were resuspended in complete RPMI-1640 and seeded in eight well Lab Teck tissue culture slides (Nunc, USA) and incubated at 37°C and 5% CO_2_ environment for 2 h for macrophage adherence. The adherent macrophages were washed (×3) with pre-warmed incomplete RPMI-1640 and infected with metacyclic promastigotes in 1:10 ratio and incubated at 37°C and 5% CO_2_ environment for 12 h. Non-phagocytized promastigotes were discarded by a simple exchange of medium and the infected macrophages were incubated with and without having the test and reference drugs (Cs-SLN, AmBisome, and AmB) in different concentrations (0.1–1 μg/ml) in complete RPMI-1640 at 37°C and 5% CO_2_-air atmosphere for 72 h. The infected macrophages were washed with PBS and stained with Wright's stain to assess the intracellular amastigote growth and intracellular amastigotes were monitored by counting at least 100 cells per slide under the oil immersion lens microscope (100 ×) (Nahar et al., 2009).

Percentage inhibition of amastigote replication was calculated by the following formula:

PI = 100 – (AT/AC) × 100
PI: percentage inhibition of amastigote multiplication,
AT: actual number of amastigotes in treated samples/100 macrophages;
AC: actual number of amastigotes in control samples/100 macrophages.

**Statistical Analysis** Origin (version 8.6) and GraphPad Prism (version 8.0.2) software were used to analyze the data of various groups of experiments. Graphs represent data from an average of 3 experiments. ^****^P-value ≤ 0.0001, ^***^P-value ≤ 0.001, ^**^P-value ≤ 0.01, and ^*^P-value ≤ 0.05 two-way ANOVA, Tukey's multiple comparisons test. The data from individual groups were presented as the mean ± SD.

## Results and Discussion

### Fabrication of Nanoparticles and Characterization

**Figure 1:**
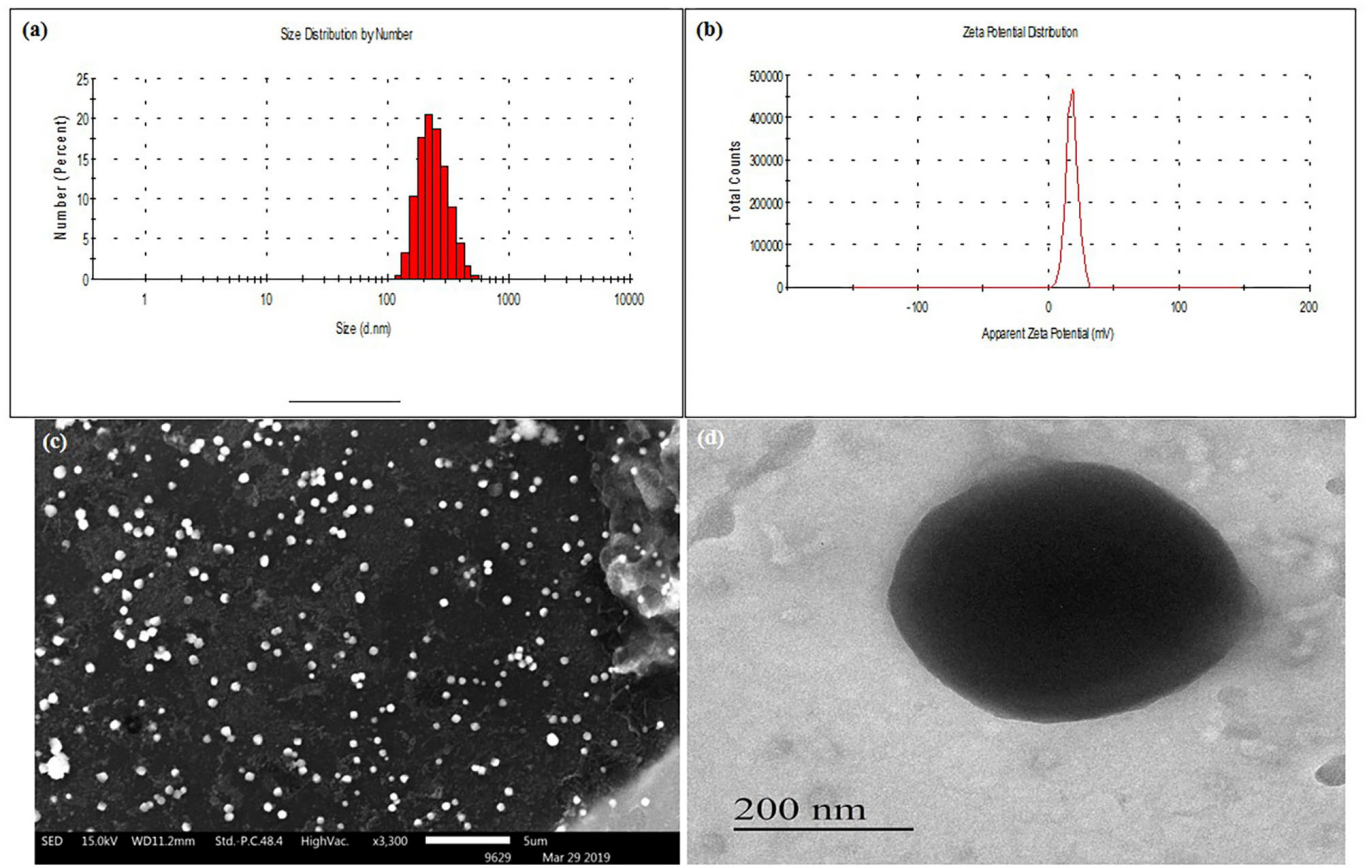
**(A)** Mean particle size (nm) of Cs-SLN. **(B)** Zeta potential (mV) of Cs-SLN. **(C)** SEM image of Cs-SLN (scale bar, 5 μm). **(D)** TEM image of Cs-SLN with 200 nm magnification.

SLN were successfully developed by using the ESE method. Formulation and process variables were optimized based on mean particle size and PDI. Optimized formulation has shown a small mean particle size of 37.6 ± 0.38 nm with a PDI of 0.31 ± 0.03 (Table 1). Nanoparticles with small size and spherical shape can be prepared by ESE (Cavallaro et al., 2015). The mean particle size of DSLN were considerably increased to 148 ± 3.60 nm with PDI 0.308 ± 0.05 and zeta potential of +1.140 ± 0.01 mV as compared to PSLN (37.6 ± 0.38 nm). Surface modifications of formulation with Cs, further increases the mean particle size to 373.9 ± 1.41 nm, respectively (Figures 1A,B). Structure as well as the morphology of Cs-SLN were verified by SEM and TEM. SLN are uniformly distributed in shape and size for both the modifications by SEM analysis 307–410 nm (Figure 1C). Surface modified formulations have shown spherical shape with a smooth surface in TEM measurements (Figure 1D). Entrapment efficiency (%) was found to be 95.20 ± 3.19% and 89.45 ± 6.86% for AmB and PM, respectively. AmB can easily encapsulated within SLN in the lipid matrix due to its lipophilic nature. Lipid-based formulation of AmB has low aqueous solubility (Battaglia et al., 2010).

**Table 1:** Particle size and zeta potential of surface-modified dual drug-loaded formulation. Results are presented as mean ± standard deviation (*n* = 3).

**Formulations**	**Mean particle**	**PDI**	**Zeta**	**AmB**		**PM**	
	**size (nm)**		**potential**				
			**(mV)**				
				**% EE**	**% DL**	**% EE**	**% DL**
PSLN	37.6 ± 0.38	0.31 ± 0.03	−7 ± 0.316	NA	NA	NA	NA
DSLN	148 ± 3.60	0.308 ± 0.05	1.14 ± 0.01	95.49 ± 2.99	23.00 ± 2.79	89.97 ± 4.06	10.5 ± 1.98
Cs-SLN	373.9 ± 1.41	0.342 ± 0.02	18 ± 0.07	95.20 ± 3.19	22.89 ± 2.67	89.45 ± 6.86	10.4 ± 1.83

### FTIR Study

**Figure 2:**
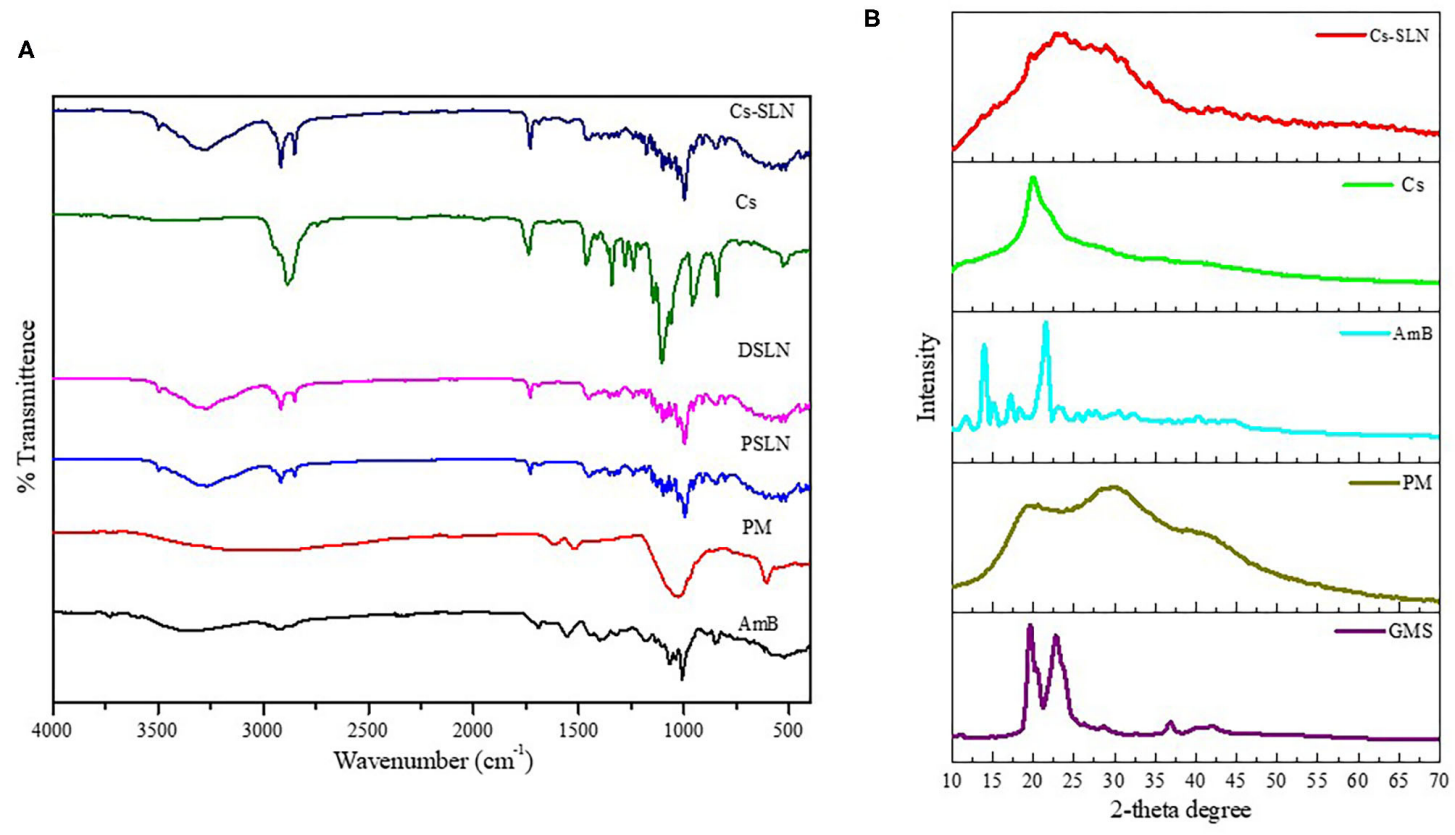
**(A)** FTIR Spectra of GMS, PM, AmB, Cs, Cs-SLN. **(B)** XRD Spectra of GMS, AmB, PM, Cs, Cs-SLN.

The characteristic peaks in FTIR spectra of GMS are present at 2,915 and 2,852 cm^−1^ (C-H stretch in the -CH_2_ groups), at 1,733 cm^−1^ (C=O stretching, fatty acid ester), at 1,108 cm^−1^ (C-O–C stretching), 3,300 cm^−1^ (O-H stretching, glycerol moiety), 1,250 cm^−1^ (C-O stretch), 1,457 and 718 cm^−1^ (C-H stretching). The FTIR spectrum showed characteristic peaks of PM at 1,534 cm^−1^ (CH_2_ bending) at 1,626 cm^−1^ (N-H bending coupled with C-N stretch), and at 1,019 cm^−1^ (C-O-C) stretch are present in spectra of PM (Khan and Kumar, 2011). The FTIR spectra of AmB showed characteristic peaks such as 3,368 cm^−1^ (-OH stretch strongly H- bond), 3,011 cm^−1^ (C-H stretch, polyene), 1,553 cm^−1^ (N-H in-plane), and 1,687 cm^−1^ (C=O stretching), 1,310 cm^−1^ (C–O stretching) shown in Figure 2A (Singh et al., 2016b). In the FTIR spectrum of Cs-SLN peaks corresponding to AmB and PM were found at 3,348 cm^−1^, 3,011cm^−1^, 1,681 cm^−1^, 1,552 cm^−1^ and 1,636 cm^−1^, 1,534 cm^−1^, respectively. There was no new peak found in the spectra of Cs-SLN which indicates the presence of drugs in dissolved form in the lipid matrix and no interaction was observed between the drugs and lipid matrix (Ghadiri et al., 2011; Butani et al., 2016).

In the IR spectra of chitosan (Figure 2A) 3,367 cm^−1^ (O-H stretching overlapping the N-H stretching), 2,867 cm^−1^ (C-H stretching), 1,637 cm^−1^ (N-H bending), 1,585 cm^−1^ (N-H stretching) 1,435–1,386 cm^−1^ (asymmetrical C-H bending of the CH_2_ group) and 1,027 cm^−1^ (C-N stretching). In the IR spectra of Cs-SLN the characteristic peaks of chitosan are present. Some peaks are shifted and broadened due to overlapping of groups and formation of nanoformulation.

### Powdered X-Ray Diffraction Study (PXRD)

PXRD was performed to determine the physical state of lipid, AmB, and PM. Lipid and AmB showed a compact and characteristic diffraction pattern. X-ray diffractogram of AmB has exhibited a sharp peak at 2θ scattered angle of 21.39, 14.1, and 21.78° indicating its crystallinity but PM was found to be amorphous in nature. Cs-SLN showed two peaks at 24.8 and 30.6° but the pattern was showing shifted, broadened, and weaker peak as compared to GMS, which was partially recrystallized and transformed to less ordered in SLN formulation. Drug peaks were also absent due to complete entrapment of drug in the lipid matrix. The less ordered and amorphous nature would be contributing to higher drug loading. Diffraction peaks broadening was related to particle sizes as the broadening of Bragg's peaks indicates the formation of nanoparticles (Figure 2B) (Kumar et al., 2016).

### Cellular Uptake Studies

**Figure 3:**
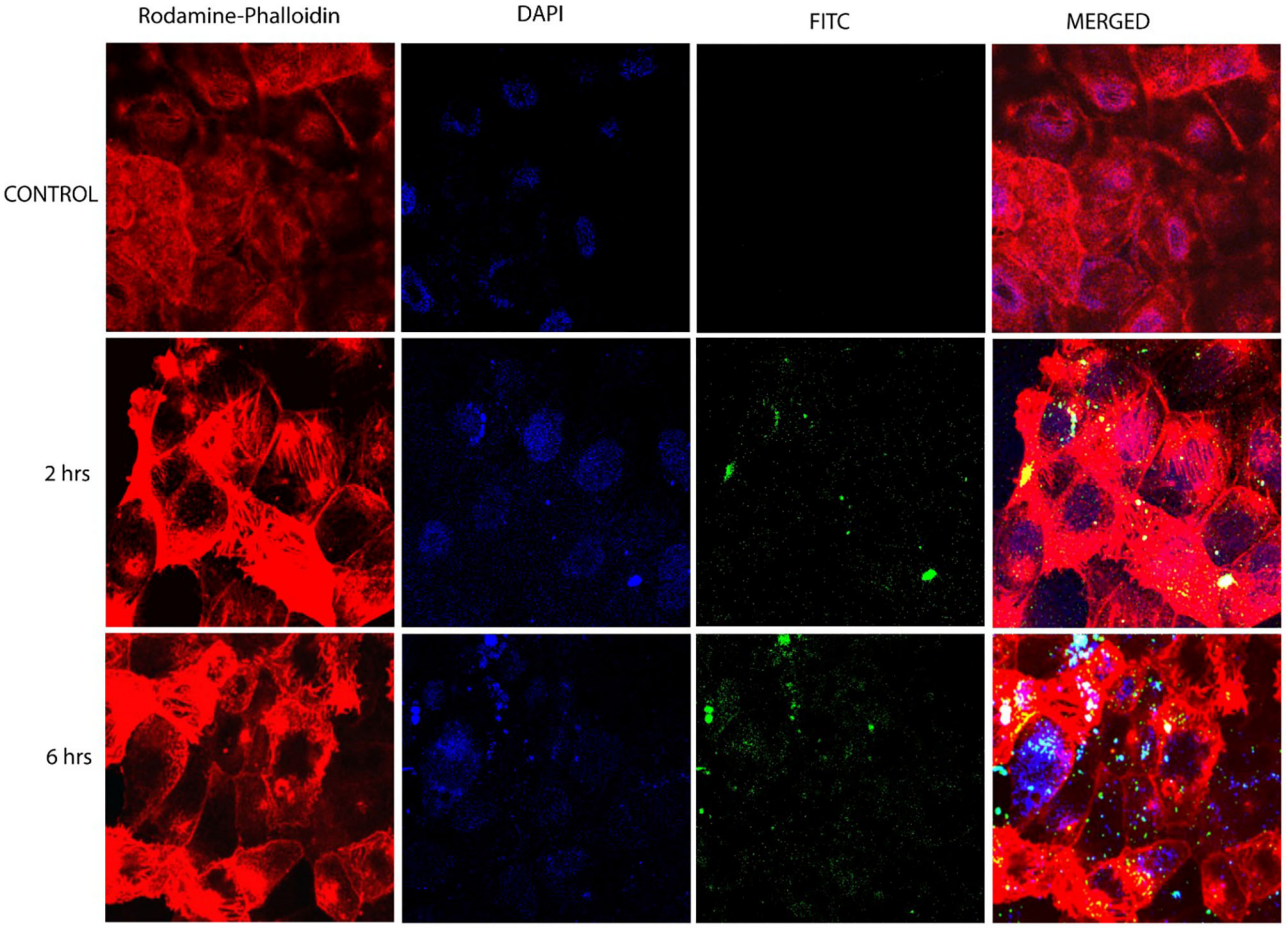
Confocal microscopy images of Caco-2 cells after 6 h incubation at 37°C with FITC-tagged Cs-SLN. FITC, fluorescein isothiocyanate; hrs, hours.

Confocal laser scanning microscopy studies were performed to FITC-tagged Cs-SLN to assess drug internalization into Caco-2 cells. It was observed that the FITC-tagged Cs-SLN were internalized by Caco-2 cells in the time-dependent way as well as no toxic response was seen because there was no change observed in the morphology of the Caco-2 cells (Figure 3). Chitosan could be used for macrophage targeting as it causes the activation of macrophages and it enhances uptake of NPs by kupffer cells (passive targeting). It was previously reported that Curcumin SLN has revealed augmented uptake in Caco2/HT-29-MTX cell monolayer up to 2 h (Guri et al., 2013). The SLN uptake efficiency can be enhanced due to surface coating (Costa et al., 2018). C6-labeled SLN and NLCs showed enhanced internalization in a time-dependent manner on HaCaT and CCC-ESF cells (Guo et al., 2015). It was observed during initial experiments that the morphology of the cells was not changed as well as normal doubling time (i.e., 48 h) even in the presence of Cs-SLN. The cells were healthy and no sign of apoptosis was observed (Vijayakumar et al., 2017). Schipper et al. (1997) reported that chitosan acts as an absorption enhancer for the poorly absorbable drugs (Schipper et al., 1997).

The higher cellular uptake could be due to the cationic surface charge of nanoparticles or might be due to the efficient binding of Cs-SLN to the cell membrane (Ma and Lim, 2003; Wang et al., 2016).

Chitosan is being accepted as a fascinating material for oral route delivery, which could be used as a permeation enhancer, mucoadhesion, and P-gp inhibition (Liu et al., 2017).

### *In vitro* Cytotoxicity Study

**Figure 4:**
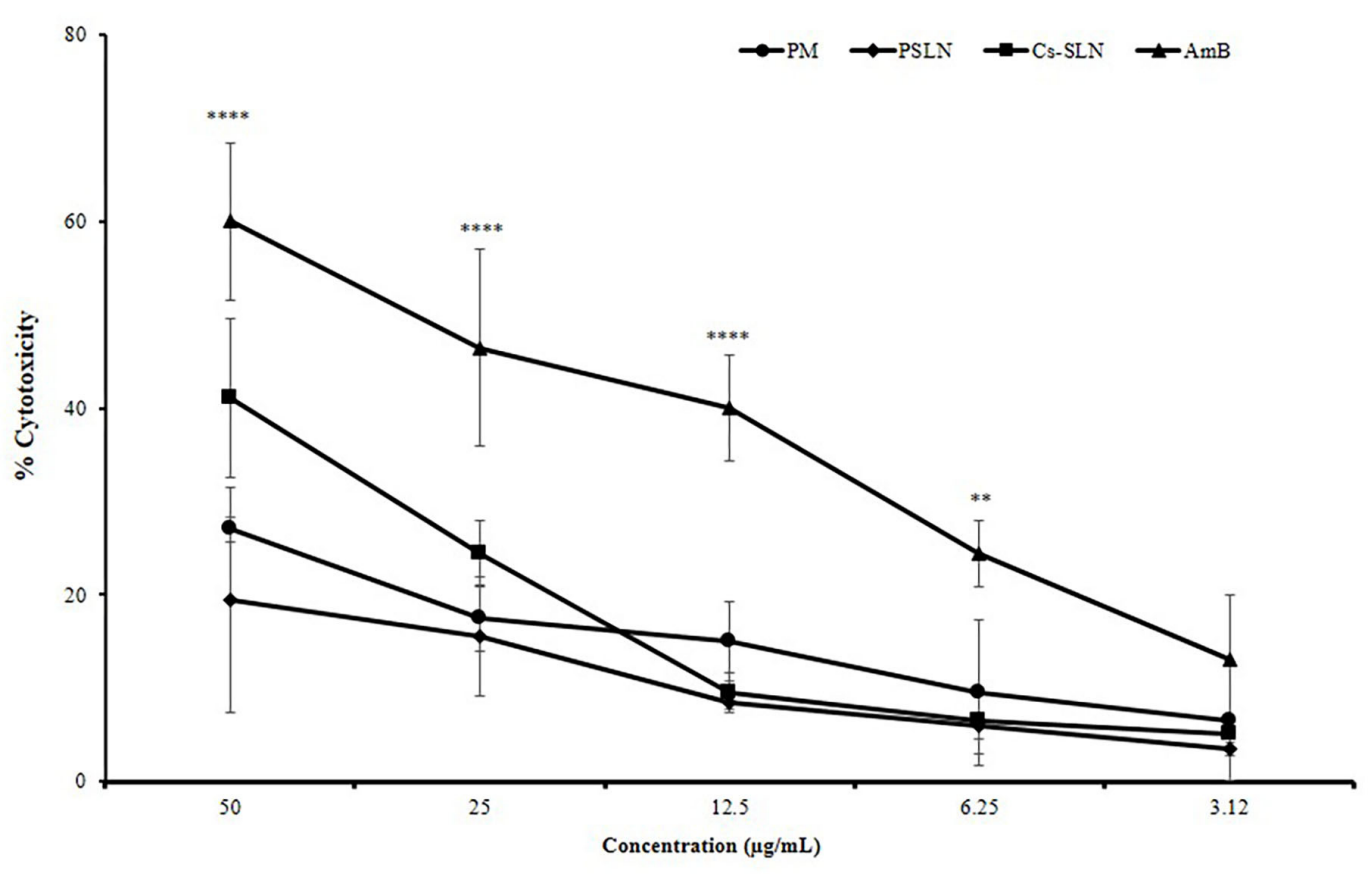
Percent cell cytotoxicity of J774A.1 cells treated with AmB suspension, PM suspension, PSLN, Cs-SLN. Results are presented as mean ± standard deviation (*n* = 3). Statistical significance was assessed using ANOVA (**p < 0.01 and ****p < 0.0001).

The cell viability is a common assay for the assessment of cytotoxicity of Cs-SLN, PSLN, PM, and AmB in macrophage cell lines (J774A.1) by MTT assay. The cell cytotoxicity of Cs-SLN, PSLN, PM were found to be less toxic when compared to free AmB, at all equivalent concentrations (Figure 4). Cell cytotoxicity study confirmed the safety of excipient used for the development of nanoparticles as the PSLN were showing more than 80% viability at all concentrations. % Cell viability reduction was found to be dose-dependent as it was observed that cell viability was lowest at 50 μg/ml of Cs-SLN. This was reflected from the controlled drug release pattern of AmB, which was showing 32.6% of drug release within 24 h. The encapsulation of AmB in SLN could be the reason for reduced toxicity and an additional surface coating with biocompatible polymers did not affect the cell viability (Ching et al., 1983). Ling et al. reported that AmB slow release from the lipid matrix could be the reason behind lower higher cell viability of Cs coated formulation (Ling et al., 2019). SLN formulations are granted as low- or non-toxic because they are produced using biodegradable compounds, which are commonly used in pharmaceuticals and cosmetics, also called GRAS (Generally Regarded As Safe) and free from the risk of acute and chronic toxicity (Bagde et al., 2019).

### *In vitro* Drug Release Study

**Figure 5:**
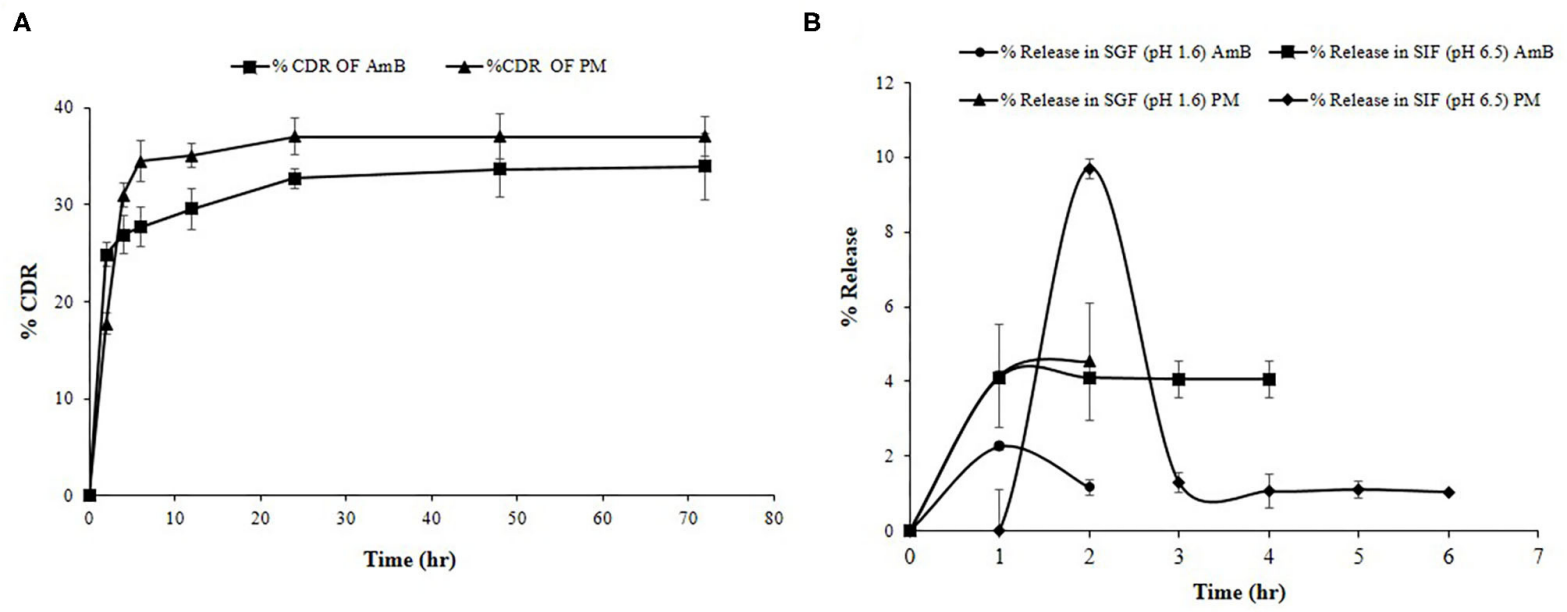
**(A)** % Cumulative drug release study of PM and AmB for Cs-SLN. **(B)** *In vitro* simulated gastric fluid drug release study of Cs-SLN. Results are presented as mean ± standard deviation (*n* = 3).

Drug release profile showed a biphasic pattern for both the drugs released from Cs-SLN (Figure 5A). Within an initial 6 h, 27.6% AmB, and 34.4% PM was found to be released due to the burst release. Being hydrophobic in nature AmB was present in the lipid core and remains inside till lipid erosion or lipid degradation process gets initiated, apart from that PM, being hydrophilic presents in the outer surfactant layer. The presence of PM on the outer layer or a little erosion of chitosan coating, might be one or both could be the reason, which leads to the higher burst release effects. Methazolamide-chitosan-SLN dispersion exhibited a biphasic release pattern, with an initial burst release about 50% within the first two hours followed by a sustained release in the following 6 h (Pozo-rodríguez et al., 2009). As observed during the drug release study % CDR (33.9%) for AmB and 37.21% for PM up to 72 h, revealed controlled and slow drug release from Cs-SLN. SLN has before now acknowledged as the controlled release drug carrier. It was previously reported that sustained drug release could be accomplished when the drug is uniformly dispersed in the lipid matrix (Almeida, 2017). Percentage drug release of carbamazepine (CBZ) was observed for a different formulation of SLN and ~66.7% of drug was released in 24 h. CBZ encapsulated in chitosan SLN showed a controlled drug release (Nair et al., 2012). Surface modification or assembly of biological polymer coating could be employed to develop sustained release characteristics of SLN. Cisplatin-loaded chitosan-coated SLN showed a significantly higher apoptosis in cancer cells, which is attributed to the increased internalization of nanocarriers and the controlled release of anticancer drugs in the intracellular environment (Wang et al., 2016).

### *In vitro* Simulated Gastric and Intestinal Fluid Study

To monitor the stability of SLN, it is very important to study the effects of gastro-intestinal fluids on the developed formulations. AmB release was found to be negligible in SGF (pH 1.6) and SIF (pH 6.5) as shown in Figure 5B. The chitosan coating prevents the premature RVT release when compared to uncoated nanoparticles (Pauluk et al., 2019). Luo and co-workers reported that the presence of chitosan coating protects the constituent drug from the effect of gastric enzymes (Luo et al., 2011). Mucoadhesive properties of chitosan enhance the interaction with the membrane as well as the absorption of compounds by the gastrointestinal tract (Farris et al., 2017).

### Mucoadhesive Property

**Figure 6:**
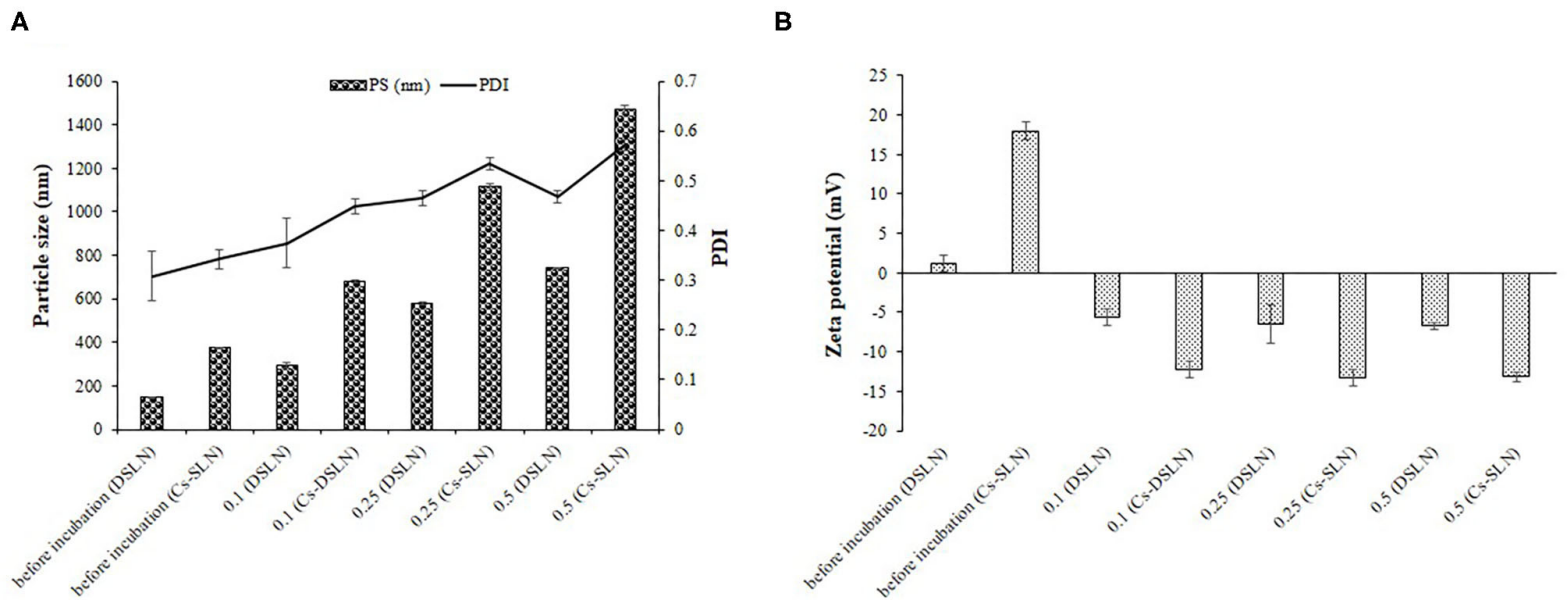
**(A)** Mean particle size, PDI of DSLN, Cs-SLN in the absence and presence of mucin. **(B)** Zeta potential of DSLN, Cs-SLN in the absence and presence of mucin. Results are presented as mean ± standard deviation (*n* = 3).

Mucin is negatively charged owing to sialic acid and Cs-SLN possessed positive charge before incubating with different mucin solutions because of the presence of an amine group. Further, a drop in the value of zeta potential was observed, which moved toward more negative, revealed the presence of interaction between the amine group of chitosan and mucin, reflecting mucoadhesive property of chitosan (Figures 6A,B) (Ling et al., 2019).

### *In vitro* Anti-leishmanial Activity of Cs-SLN Against *L. donovani*-Infected J774A.1 Macrophages

**Figure 7:**
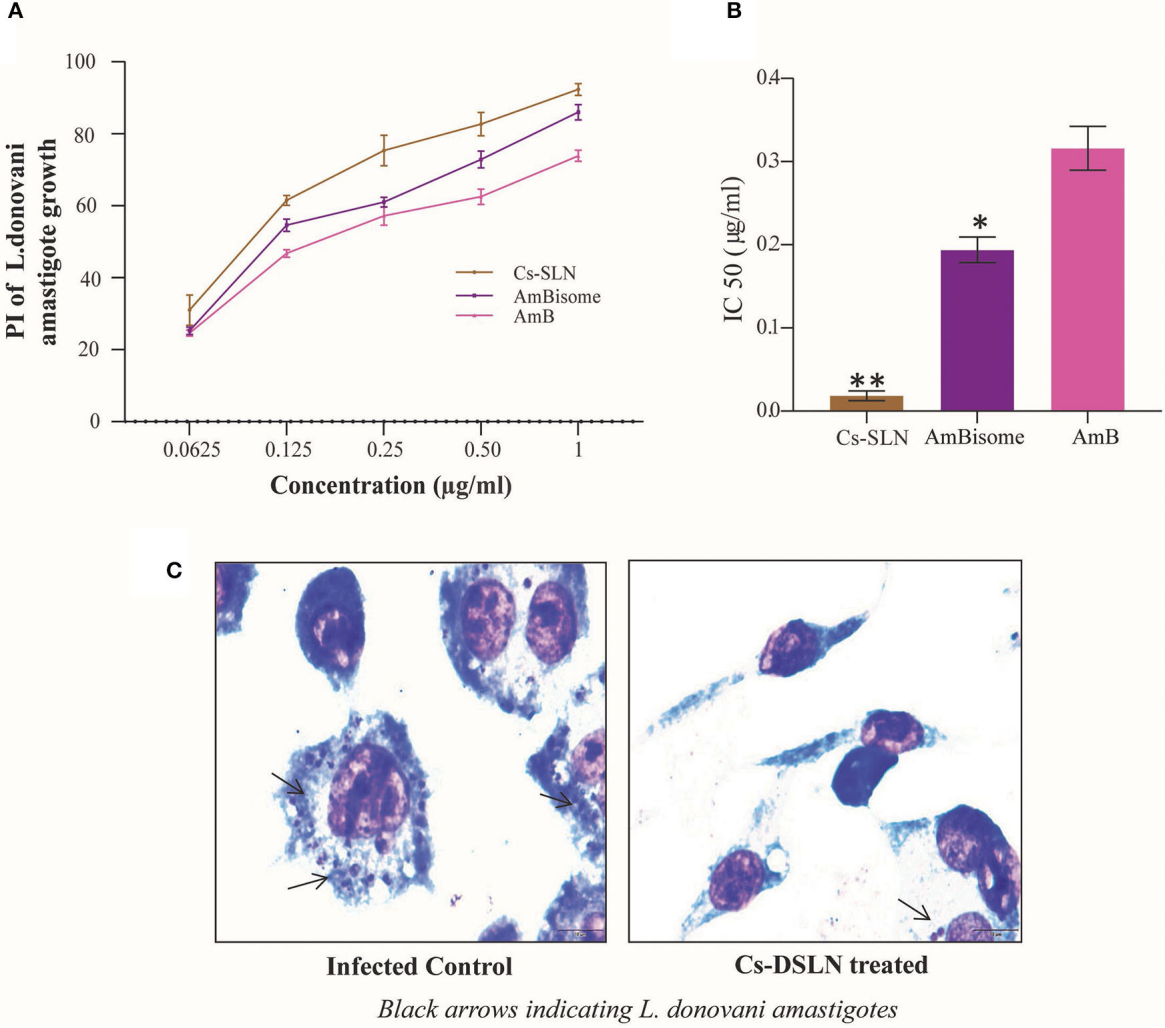
**(A)** Percentage inhibition of Cs-SLN, AmBisome, and AmB against *L. donovani*-infected J774A.1 macrophages. **(B)** IC_50_ values of Cs-SLN, AmBisome, and AmB against *L. donovani*-infected macrophages. **(C)** Microphotographs of Cs-SLN treated and untreated *L. donovani*-infected J774A.1 macrophages. Results are presented as mean ± standard deviation (*n* = 3) and analyzed by Graph Pad Prism 8.0.2 software. (Cs-SLN and AmBisome) vs. AmB, **P < 0.01, *P < 0.05.

Anti-leishmanial activity of Cs-SLN, AmBisome, and AmB were evaluated against *L. donovani*-infected macrophages, *in vitro*. All samples were stained and intra-cellular amastigotes were enumerated. Cs-SLN (1 μg/ml) have significantly (*P* < 0.01) diminished the intra-cellular amastigotes compared to free AmB (Figure 7C). The IC_50_ values of Cs-SLN, AmBisome, and AmB were observed to be 0.018422 ± 0.005928 μg/ml, 0.193894 ± 0.015368 μg/ml, and 0.316039 ± 0.026423 μg/ml, respectively (Figure 7B). The IC50 value of Cs-SLN, was significantly lower than AmBisome (*P* < 0.05) and AmB (*P* < 0.01). The IC50 value of Cs-SLN was 17.5- and 10.7-fold lower than the IC50 value of free AmB and Ambisome, respectively. The enhanced anti-leishmanial activity in macrophages is due to surface modification with Cs, which could be accountable for enhanced macrophage internalization. Cs can withstand the lysosomal pH owing to its acid resistive nature. Cs is also known to target macrophages via glucosamine-like receptors displayed on the surface of kupffer cells, the resident liver macrophages. Moreover, chitosan being positively charged interact with cell membranes easily, possessing overall negative charge therefore expedites endocytosis (Singh et al., 2016b). Cs-SLN (1 μg/ml) showed a maximum percentage of inhibition (92.35%) on intra-cellular amastigote growth of *L. donovani* (Figure 7A). Cs coating of SLN activates macrophages to release Th 1 cytokines which in turn generates an enhanced immunological response thereby facilitating the elimination of intracellular parasites (Jain et al., 2014) and AmB, a gold standard for leishmaniasis could be orally delivered by utilizing lipid-based nanocarriers with reduced adverse effect (Thanki et al., 2019).

## Conclusion

Cs-SLN was successfully developed by the emulsion solvent evaporation method and well-characterized by FTIR, NMR, XRD, SEM, and TEM analysis. Cs-SLN has shown complete internalization in Caco-2 cells. Cs-SLN has shown a sustained drug release profile over a period of 72 h and stable at gastric fluids, confirmed by simulated gastro-intestinal fluids study. Cs-SLN (1 μg/ml) have significantly (*P* < 0.01) reduced the intracellular amastigotes compared to free AmB without showing any toxic side effects. Additionally, surface modification could provide higher macrophage targeting and divulge an immunotherapeutic activity.

## Data Availability Statement

All datasets generated for this study are included in the article/supplementary material.

## Author Contributions

SP, GY, AK, OS, AV, SS, and SM: Methodology, Software, Data curation, Writing—Original draft preparation, Visualization, Investigation, Writing—Reviewing and Editing. SM: conceptualized the idea, acquired the funding, supervised the research work. All authors contributed to the article and approved the submitted version.

## Conflict of Interest

The authors declare that the research was conducted in the absence of any commercial or financial relationships that could be construed as a potential conflict of interest.

## Footnotes

^1^https://www.who.int/news-room/fact-sheets/detail/ leishmaniasis, 2019.
